# Design of surface nanostructures for chirality sensing based on quartz crystal microbalance

**DOI:** 10.3762/bjnano.13.100

**Published:** 2022-10-27

**Authors:** Yinglin Ma, Xiangyun Xiao, Qingmin Ji

**Affiliations:** 1 Herbert Gleiter Institute for Nanoscience, School of Materials Science and Engineering, Nanjing University of Science and Technology, 200 Xiaolingwei, Nanjing, 210094, China

**Keywords:** assembled nanostructure, chiral surface, chirality recognition, quartz crystal microbalance (QCM), sensing applications, surface architecture

## Abstract

Quartz crystal microbalance (QCM) has been widely used for various sensing applications, including chirality detection due to the high sensitivity to nanogram or picogram mass changes, fast response, real-time detection, easy operation, suitability in different media, and low experimental cost. The sensing performance of QCM is dependent on the surface design of the recognition layers. Various strategies have been employed for studying the relationship between the structural features and the specific detection of chiral isomers. This review provides an overview of the construction of chiral sensing layers by various nanostructures and materials in the QCM system, which include organic molecules, supermolecular assemblies, inorganic nanostructures, and metal surfaces. The sensing mechanisms based on these surface nanostructures and the related potentials for chiral detection by the QCM system are also summarized.

## Introduction

Chirality is a prevalent phenomenon in nature. Many common biological macromolecules such as proteins, ribose, and cellulose are inherently chiral. Chiral molecules have two forms (enantiomers) that are mirror images of each other. It is a mysterious phenomenon that almost all chiral molecules in living organisms are found in one form, such as right-handed sugars, left-handed amino acids, and right-handed DNA coils. Scientists coined the term “homochirality” to describe single-handedness as a prerequisite for all molecules present in living organisms, which might also be the initial statement for chiral selection [1–4]. Therefore, discrimination of enantiomers is extremely vital for understanding the biological functions and studying the mysteries of life [5–6].

Chiral enantiomers have identical molecular formulas and the same physical properties. However, they can exhibit completely different functions. One isomer form may act as a powerful medicament, while the other might cause serious side effects. The famous "thalidomide disaster" in the 1960s affected several pregnant women causing fetal malformation [7], owing to the trace presence of teratogenic (*S*)-thalidomide in sedative (*R*)-thalidomide [8]. Hence, chirality detection keeps attracting much attention in the fields of chemistry, biology, and medicine. Especially for the pharmaceutical and food industry, detection and separation of enantiomers are essential for their safe usage.

With appropriate detectors and transduction signals, various analysis instruments and techniques have been used for chirality detection or chiral separation. They include circular dichroism (CD) [9], nuclear magnetic resonance [10], Fourier transform infrared spectrometry (FTIR) [11], UV–vis absorption spectrometry [12], mass spectrometry (MS) [13], titration microcalorimetry [14], high-performance liquid chromatography (HPLC) [15], gas chromatography (GC), capillary electrophoresis (CE) [16], and electrochemical chiral sensors [17–19]. However, these methods still have drawbacks as they are time consuming, expensive, and unable to monitor real-time detection. In addition, they have low-recognition efficiency and low sensitivity to weak signals. Therefore, researchers keep devoting their efforts to developing novel sensing systems for enantiomers, which still remains challenging.

Quartz crystal microbalance (QCM) is a well-known mass-sensor technique capable of recording changes in nanogram or even picogram levels in both gas and liquid phases [20–21]. The sensing of mass changes is based on the oscillation frequency of an AT-cut quartz crystal resonator, which may cause a decrease in frequency when the analyte is adsorbed on the surface [22]. Due to its easy operation, low cost, compactness for lab-on-chip usage, good stability, and real-time recording, QCM has been widely used for various biological analyses (e.g., DNA analysis, microorganism assays, nucleic acid detection, pharmaceutical substance detection, and gas monitoring) and also a powerful tool for chiral recognition [23–25].

The sensitivity and specificity of QCM-based chiral sensors largely depend on the recognition layers on the surface of the quartz crystal resonator [26]. During the sensing process, the guest molecule may selectively bind to host molecules such as the “lock and key” model. This type of “host–guest” recognition requires high-level molecular compatibility of the selectors and the analytes. The driving forces are mostly based on noncovalent interactions, including hydrogen bonding, metal coordination, van der Waals forces, π–π interaction, and electrostatic interaction*.* Moreover, the structural “fitting” effect may also have distinct adsorption behaviors for enantiomers. Therefore, the design of effective chiral sensors is always based on aspects of effective chiral host molecules, proper chiral surface functions, and suitable host nanostructures. To achieve high chiral selectivity and sensitivity, it is also essential to understand the basis of the interactions for the formation of transient diastereomeric complexes by analytes and chiral hosts in the recognition processes. Basically, the host selectors need to have chirality to ensure specific recognition of analytes. The chirality of the host layers may derive from intrinsic chiral molecules/substrates using chiral templates, chiral modifications, and induced by external stimulus.

As there are almost no limitations for the receptor layers in QCM sensor systems, various materials and nanostructures have been developed for constructing sensing layers on the surface of the electrode. The sensing process may also be implemented in the liquid and gas phases. These make QCM a versatile platform for both constructing novel sensing systems and studying sensing behaviors, whose advantages are incomparable to other conventional chiral sensing systems. In this review, we briefly summarized a wide range of nanostructures for chiral sensing based on QCM systems, including biomolecular layers, chiral molecular assemblies, polymer hybrids, inorganic nanostructures, and metal crystals.

## Review

### Construction of molecular layers for chiral sensing by QCM

Organic molecular layers are the most common selectors used for chiral recognition in the QCM system. The selector layers, composed of biomolecules, polymers, supramolecular assemblies, and functional organic hybrids have been successfully constructed on the surface of QCM electrodes. According to frequency shifts of QCM upon adsorption, detection efficiencies of various selectors for chiral analytes could be evaluated. The recognition mechanisms may also be studied by combining QCM with other techniques.

#### Sensing layers by chiral biomolecules

As chiral recognitions are fundamental phenomena in biology, biomolecules of amino acids, proteins, and nucleic acids are ideal chiral selectors. They have been extensively employed in various chiral sensing applications for the study of biological recognition processes [27]. The biomolecule-based selectors discriminate enantiomers of target analytes via their complementarity. This means that the target should energetically and structurally fit with the receptor.

**Figure 1:**
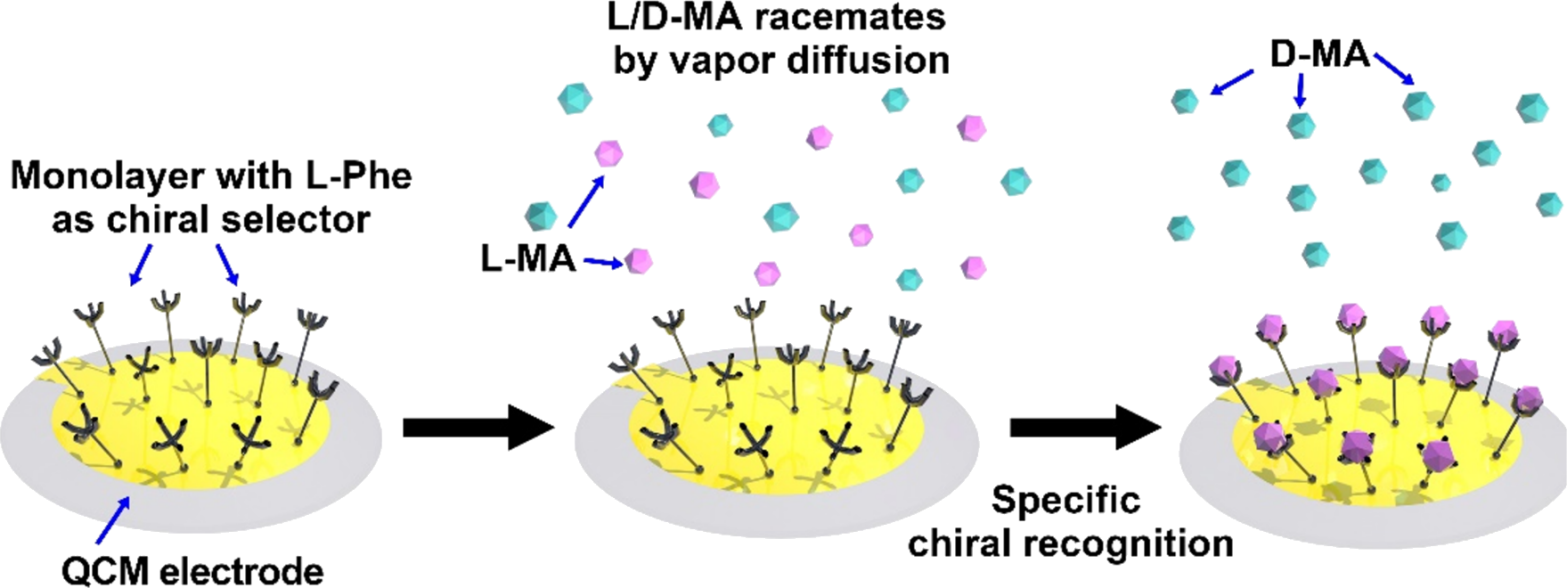
Self-assembly monolayer of ʟ-phenylalanine as a selector layer for the QCM chiral sensor [26].

Amino acids are a type of simple natural molecule which is widely applied for the specific recognition of chiral analytes in QCM. The molecular layers of amino acids may be immobilized on the surface of electrodes by chemical modification and self-assembly. Mandelic acid (MA) is an important chiral intermediate in the pharmaceutical industry. It has been taken as a model molecule to verify the “proof-of-principle” concept for chiral recognition [28]. Kim and co-workers fabricated ʟ-phenylalanine (ʟ-Phe)-modified QCM sensor and used vapor diffusion molecular assembly (VDMA) to study the chiral adsorption of MA (Figure 1) [26]. The immobilization of ʟ-Phe on QCM sensors was through a two-step assembling process. A 4-aminothiophenol (ATP) monolayer was firstly adsorbed on the QCM surface by chemical Au–S bonding. Then, 4-NH_2_-ʟ-Phe was bound by a diazo-coupling reaction to form a monolayer with ʟ-Phe. The adsorptions of ʟ-MA, ᴅ-MA, and ʟ/ᴅ-MA racemates on the ʟ-Phe sensing layers was monitored through the evaporation diffusion of the solutes. The QCM results indicated that the ʟ-Phe-modified sensor surface had selective chiral recognition ability to MA. Through the control of different VDMA periods and adsorption comparison between deionized H_2_O, ᴅ-MA, and the racemate of MA, it was revealed that the specific recognition should be ascribed to the hydrogen bonding between the immobilized ʟ-Phe and the analyte ʟ-MA.

Valine (Val) is one of the eight essential amino acids of the human body, which plays a crucial role in various biological processes. Since its isopropyl side group may connect to the chiral center, Val may be an ideal model to study the influence of hydrophobic side groups on a possible chiral effect. Sun et al. investigated the chiral adsorption of different modulate proteins on ʟ(ᴅ)-Val modified QCM sensors [29]. The sensor layers were constructed by self-assembly of ʟ(ᴅ)-Val monolayers or comb-teeth-type grafted polymer brushes with ʟ(ᴅ)-Val. According to the frequency shifts upon bovine serum albumin (BSA) adsorption, ʟ-Val-modified sensors were shown to have a stronger binding to BSA. A similar QCM response is also seen for gelatin. As physicochemical properties of BSA and gelatin are different, it was inferred that the chiral sensing effect of this system could be applied to various protein species. The authors further employed fluorescent titration measurements to study the affinity between proteins and chiral selectors. It was elucidated that stereoselective hydrophobic interactions are the major driving forces that govern protein adsorption on ʟ-Val-modified surfaces.

Besides small biomolecules, biological macromolecules, such as proteins and enzymes, also show natural specific recognition and selective functions in enzymatic reactions and metabolic processes [30]. Therefore, they may also be suitable as chiral selectors in QCM sensing systems. Zhang and Ng et al. successfully fabricated rapid and real-time QCM biosensors based on self-assembled monolayers (SAMs) of serum albumin (SA) [31]. They evaluated the detection efficiencies of ten enantiomers, which were tetrahydronaphthylamine (TNA), 1-(4-methoxyphenyl)ethylamine (4-MPEA), 1-(3-methoxyphenyl)ethylamine (3-MPEA), 2-octanol (2-OT), and methyl lactate (MEL). The fixation of BSA and human serum albumin (HSA) layers on the QCM surface was done through the pretreatment of mercaptoacetic acid on the QCM surface. The free carboxyl groups were then linked to SAs by a coupling agent to form SAMs. The QCM results indicated that BSA- and HSA-modified QCM sensors have a specific recognition ability, especially for detecting TNA and 4-MPEA enantiomers. The chiral recognition was probably driven by the compatibility of chiral molecules with their three-dimensional structures and noncovalent interactions.

Zhang et al. developed a class of QCM chiral sensors with SAMs of BSA, HSA, goat serum albumin (GSA), or rabbit serum albumin (RbSA) for real-time chiral recognition of five pairs of enantiomers [32]. Although SAs of different species have similar structures with 70–80% identical amino acid sequences, they showed different chiral recognition abilities. For example, the maximum chiral recognition degree α_QCM_ of the GSA layer was 1.34 for *R*/*S*-1,2,3,4-tetrahydro-1-naphthylamine (*R*/*S*-1-TNA), while RbSA was 1.28. The recognition preference for different enantiomers also showed to be opposite to GSA and RbSA. 2-Octanol (2-OT) and methyl lactate (MEL) showed higher binding strength to the *S*-form on GSA and RbSA selector layers, which was opposite to that of 1-TNA and 1-(3-methoxyphenyl)ethylamine (3-MEPA). Combined with ultraviolet (UV) and fluorescent (FL) analysis results, these results indicated that the differences in chiral recognition are caused by diverse molecular interactions between enantiomers and SAs. 1-TNA and 3-MPEA may induce the SA peptide chain to stretch and the aromatic heterocyclic hydrophobic groups of Trp residues and Tyr residues to be exposed, which weaken hydrophobic interactions and strengthen n–π* and π–π* interactions. For 2-OT and MEL cases, their hydroxy groups (−OH) may form typical hydrogen bonds with the amino acid residues of SA molecules.

Based on the QCM responses of naproxen (Nap) recognition on the BSA selector layer, Guo et al. studied the chiral adsorption forces by cyclic voltammograms (CVs) [33]. The result showed the formation of a larger electron transfer blocking layer between *R*-Nap and BSA. This suggested that this stronger interaction with *R*-Nap rather than with *S*-Nap should arise from different steric hindrance effects between BSA and *R*/*S*-Nap. The result was consistent with the QCM measurements.

#### Polymer-based films for chirality sensing

Polymers have been widely used as matrix layers to physically or chemically bind with recognition molecules for chiral detection [34–35]. Compared with SAMs, polymer-supported recognition molecules may strengthen chiral bindings and detection efficiency. Gao et al. studied the Val enantiomer-containing polymer, poly(acryloyl-ʟ(ᴅ)-valine) (ʟ(ᴅ)-PAV), as the chiral recognition layer for detection of ʟ-lecithin [36]. They found that ʟ(ᴅ)-PAV possesses larger chirality than the valine enantiomer-containing monolayer of 2-mercaptoacetyl-ʟ(ᴅ)-valine (ʟ(ᴅ)-MAV). The frequency responses by QCM indicated that ʟ-lecithin adsorbs more rapidly on ʟ-PAV, but has a larger final adsorption amount on ᴅ-PAV. Based on isothermal titration calorimetry (ITC) analysis, they proved that both ʟ- and ᴅ-PAV thermodynamically favored the binding of ʟ-lecithin and ᴅ-PAV had a stronger affinity for ʟ-lecithin.

Yang et al. synthesized serine derivatives based on homochiral coordination polymers (HCPs) of (ʟ)/(ᴅ)-SA-Cd and used them as enantioselective sensors toward guest enantiomers [37]. According to QCM measurements, the enantioselective factor values for lactic acid, menthol, valinol, and ʟ-phenylethylamine (PEA) were 1.72 ± 0.15, 1.81 ± 0.08, 1.37 ± 0.03, and 2.89 ± 0.09, respectively. Through further electrochemical tests, HPLC analysis, and theoretical calculations it was revealed that ʟ- and ᴅ-forms of SA-Cd exhibited mirror behaviors towards guest enantiomers, and HCP construction may enhance enantioselectivity. The oriented H-bonding between the chiral –OH groups of serine and –NH_2_ of PEA was the binding force for enantioselective recognition.

**Figure 2:**
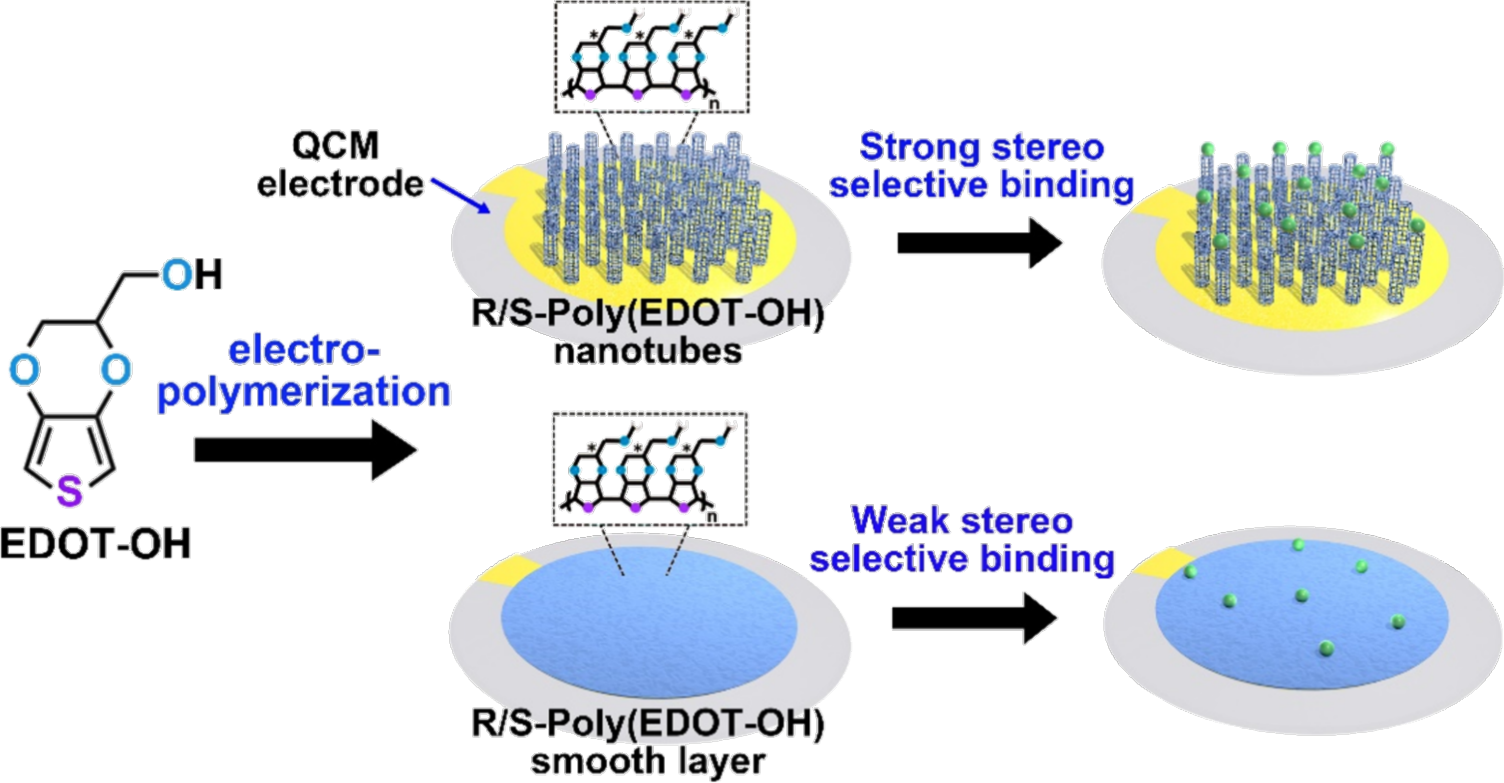
Poly(EDOT-OH) layers with different morphologies for chiral detection in the QCM system. Redrawn from [38].

Yu et al. designed new template-free polymer films based on the electropolymerization of 3,4-ethylenedioxythiophene monomers (EDOT) with an –OH functional group for chiral recognition of biomolecules (Figure 2) [38]. The sensing films of poly(EDOT-OH) with either *R* or *S* chirality were directly synthesized on the surface of the QCM electrode and engineered with different morphologies of nanotubular arrays and smooth membranes. The binding effects of fetal bovine serum, RGD peptide, insulin, and mandelic acid were examined by QCM. The results indicated that the molecules stereoselectively bound on the films and have a greater affinity toward the nanotubular film than toward the smooth film. This proved that the structural features of the polymer sensor film are also key factors for improving the chiral recognition efficiency.

**Figure 3:**
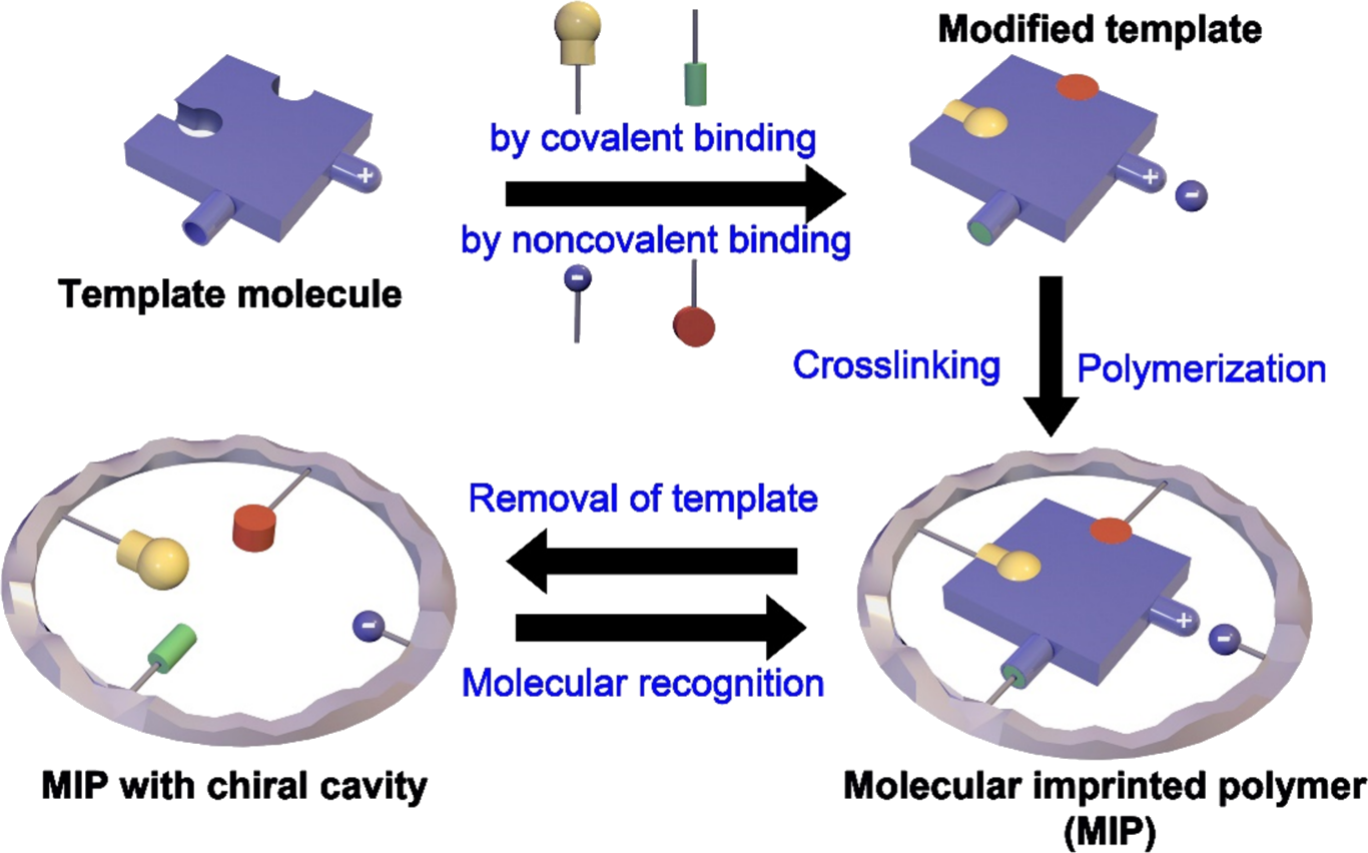
The formation of molecular imprinted polymers for chiral recognition [39].

Although polymer-supported chiral moieties showed enhanced sensing capability of chiral molecules, this type of complex mode still has stability problems. The recognition moieties in polymer layers may detach or denature during the chiral sensing process or long-time usage, which could cause a deviation or decrease in the sensing performance. To avoid these unfavorable changes in chiral sensing, researchers developed a specific recognition strategy called molecular imprinting technology (Figure 3) [39–40]. In this strategy, the chiral template molecules are first mixed with monomers. Polymerization is then induced to form molecularly imprinted polymers (MIPs). Finally, the immobilized chiral templates are eluted, leaving imprinted cavities for chiral recognition [41]. According to the shape, size, and functional groups of the cavities, MIPs can achieve specific binding with high detection efficiency to certain chiral molecules. Besides the analytes with the same structure as the imprinted template molecules, specific recognition sites left in the cavity may also detect analytes with completely different imprinted structures [42–43]. These recognition modes could enable sensing even in a complex environment with harsh conditions, such as high temperature, acidity, or alkalinity [44].

Haupt and Kutner et al. embedded the β-blocker *S*-propranolol within a poly(trimethylolpropane trimethacrylate-*co*-methacrylic acid) membrane on the surface of QCM electrodes [45]. After removal of the template by acetic acid–acetonitrile solution, permeable MIP-sensing films with an imprinted cavity could be obtained. The real-time adsorption measurements indicated that this MIP film exhibited specific recognition only for *S*-propranolol.

Luo et al. used the in situ bulk polymerization method to fabricate poly(methacrylic acid) (PMAA) MIP films on QCM electrodes for detecting ʟ-tryptophan. The sensor film was highly selective with a detection limit of 0.73 ng/mL and did not respond to the structural analogous of ᴅ-tryptophan and ascorbic acid. The selectivity coefficient of ʟ/ᴅ-tryptophan on the MIP film could be as high as 7.23. The proposed MIP–QCM sensor was also successfully applied to determine the amount of tryptophan in real sample analysis for food and urine samples with an excellent recovery of 97–104% [46].

Cao et al. fabricated methacrylic acid- (MAA) and 4-vinylpyridine- (4-Vpy) based polymer film with dansyl-ʟ-phenylalanine as the chiral template. The template was finally removed by washing with a solution of 0.01 M HCl. The resultant MIP could specifically recognize dansyl-ʟ-phenylalanine in solution and quantitatively measure ʟ- and ᴅ-forms in the enantiomer mixture [47]. Based on the QCM system, they also studied the relationship between the recognition efficiency and the number of chiral cavities in MIP films, which was achieved by varying the concentrations of the chiral template (dansyl-ʟ-phenylalanine) during the MIP formation process. The results indicated that a high addition of the chiral template in the MIP film decreased the recognition capability. At higher concentrations, the chiral template molecules form clusters and leave larger cavities in MIP films, which does not favor the dimensional recognition of the target enantiomer. As the intermolecular interactions with the analyte also play a role in chiral recognition, the group also studied the influence of pH values. The highest recognition efficiency was shown at pH 10 since basic conditions may promote the formation of stronger hydrogen bonding between the carboxyl group of the analyte and pyridyl sites in MIPs.

The MIP-based selectors on the QCM surface can be achieved not only by in situ polymerization but also by using premade MIP nanoparticles. Krozer et al. reported the fabrication of QCM chiral sensors by physically entrapping MIP nanoparticles into a spin-coated poly(ethylene terephthalate) (PET) layer on the surface of an electrode [48]. By controlling the deposition conditions, a stable layer with a high loading amount of MIP nanoparticles could be obtained, which would allow for the detection limit of propranolol to be 2 nmol·cm^−2^ or approx. 1 × 10^15^ molecules·cm^−2^. The chiral discrimination between *R*- and *S*-propranolol can also be achieved.

**Figure 4:**
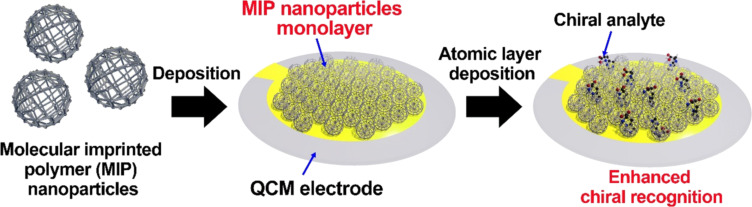
The chiral selector layer fabricated by the deposition of molecular imprinted polymer nanoparticles with enhanced detection efficiency in the QCM system [49].

Sönmezler et al. prepared ʟ-histidine-imprinted poly(EGDMA-MAH/Cu(II)) nanoparticles with a size of 86.43 nm to construct QCM sensors (Figure 4) [49]. The thickness measurements demonstrated that the particle films were almost forming a monolayer on the surface of the QCM electrode. Compared with the nonimprinted polymer nanoparticle (NIP) film, MIP nanoparticle films displayed higher adsorption ability for ʟ-histidine, which was approx. 5.8 times higher than that for ᴅ-histidine and 2.2 times higher than that for ʟ-tryptophan.

Ye et al. studied the influence of MIP film thickness on the chiral recognition of *R*/*S*-propranolol [50]. The MIP film was composed of poly(methacrylic acid) with *S*-propranolol as the chiral template. From the frequency shifts of QCM, the thinner film was shown to facilitate the exposure of recognition sites which may increase the chiral sensing efficiency of *S*-propranolol.

Besides chiral detection of the analyte by imprinting the same molecule, chiral recognition may also be performed in MIP films fabricated with different chiral templates. Kaner et al. reported chiral recognition of ᴅ/ʟ-phenylalanine in polyaniline (PANI) film using *R*-camphorsulfonic acid (*R*-CSA) as the chiral template [51]. The QCM measurements indicated that the CSA-depleted PANI film showed a preference not only for adsorption of ᴅ-phenylalanine but also had similar responses for ᴅ-alanine and ᴅ-glutamate. Combined with color changes and UV–vis spectra of the sensing solutions, the driving force for the chiral detection was mainly suggested to be due to the induced chirality of PANI film by *R*-CSA.

#### Chiral recognition layer from supermolecular structures

Supermolecular structures are self-assembled structures formed by noncovalent intermolecular/intramolecular interactions of hydrogen bonds, electrostatic, van der Waals, and hydrophobic interactions [52–54]. As the construction concept is inspired by natural systems for molecular recognition, supermolecular-based nanostructures have attracted intensive attention in various applications and also show great potential for selective separation and sensing applications [55].

Through adjusting building blockings, sensing hosts of supermolecular structures with different channel sizes, shapes, and functional sites could be fabricated, which provides highly specific recognitions or responses for chiral analytes [56–57]. They may allow for systematic molecular level detection helping to elucidate recognition behaviors with various structural features [58–59]. There are various strategies to construct supermolecular sensing films on the surface of QCM electrodes for chiral sensing.

**Cyclodextrin derivatives.** Cyclodextrins (CDs) are a class of oligomers composed of glucose units. They possess hollow truncated cones with a lipophilic inner cavity and a hydrophilic edge. The hydroxy groups at the edge can undergo different chemical reactions, which allows for modifications by a variety of functional substituents [60–62]. Therefore, CDs and derivatives are the most used host superstructures to adapt the selection of various chiral guests. The chiral recognition sites of CDs may arise from their glucose chiral units and functionalities on the rims. The key to the chiral recognition by CDs is the formation of diastereomeric host–guest complexes based on different interaction affinities. The complexation may occur via inclusion of the guest chiral molecule into the cavity of CDs by π–π interactions, dipole–dipole, ion-pairing, hydrogen bonding, and electrostatic and steric repulsion interactions, which may cause subtle structural changes to be detected [63–65]. The functional substituents on the CD rims also play an important role in the chiral recognition process, which may generate driving forces more efficiently and provide a higher interaction energy for the host–guest complexes to discriminate the enantiomers in the inclusion process [66].

Fietzek et al. measured the selective adsorption of chiral limonene in three different β-cyclodextrin (β-CD) derivatives by QCM and artificial neural networks (ANN) to evaluate the chiral discrimination performance [67]. It was revealed that better chiral discrimination was achieved with heptakis(2,3-di-*O*-methyl-6-*O*-*tert*-butyldimethylsilyl)-β-cyclodextrin and heptakis(2,3-di-*O*-ethyl-6-*O*-*tert*-butyldimethylsilyl)-β-cyclodextrin (Me- and Et-β-CD) than that with heptakis(2,3-di-*O*-acetyl-6-*O*-*tert*-butyldimethylsilyl)-β-cyclodextrin (Ac-β-CD). Combined with gas chromatography analysis, chiral separation factors could be estimated. Et-β-CD showed a more sensitive QCM response to *R*-limonene. It was also found that a higher chiral recognition factor was gained at the lowest limonene concentration due to more recognition sites in CDs exposed for preferential adsorption of *R*-limonene.

Ng et al. synthesized thiol-functionalized cyclodextrins with different linker lengths via the Staudinger reaction for chiral recognition of *R*/*S*-lactate methyl ester [68]. Through a strong reaction between the thiol group and gold, CD films with different network densities, according to the linker lengths, were constructed on the surface of the QCM electrode. The film density changed with the sulfhydryl linker length which may influence the detection performance. The chiral differentiation factor for *R*/*S*-methyl lactate may increase with the density of the modified-CD film. The longer linkers may concentrate the CDs in the film, favoring the interactions between the inner cavity of the CDs and the chiral analytes.

**Figure 5:**
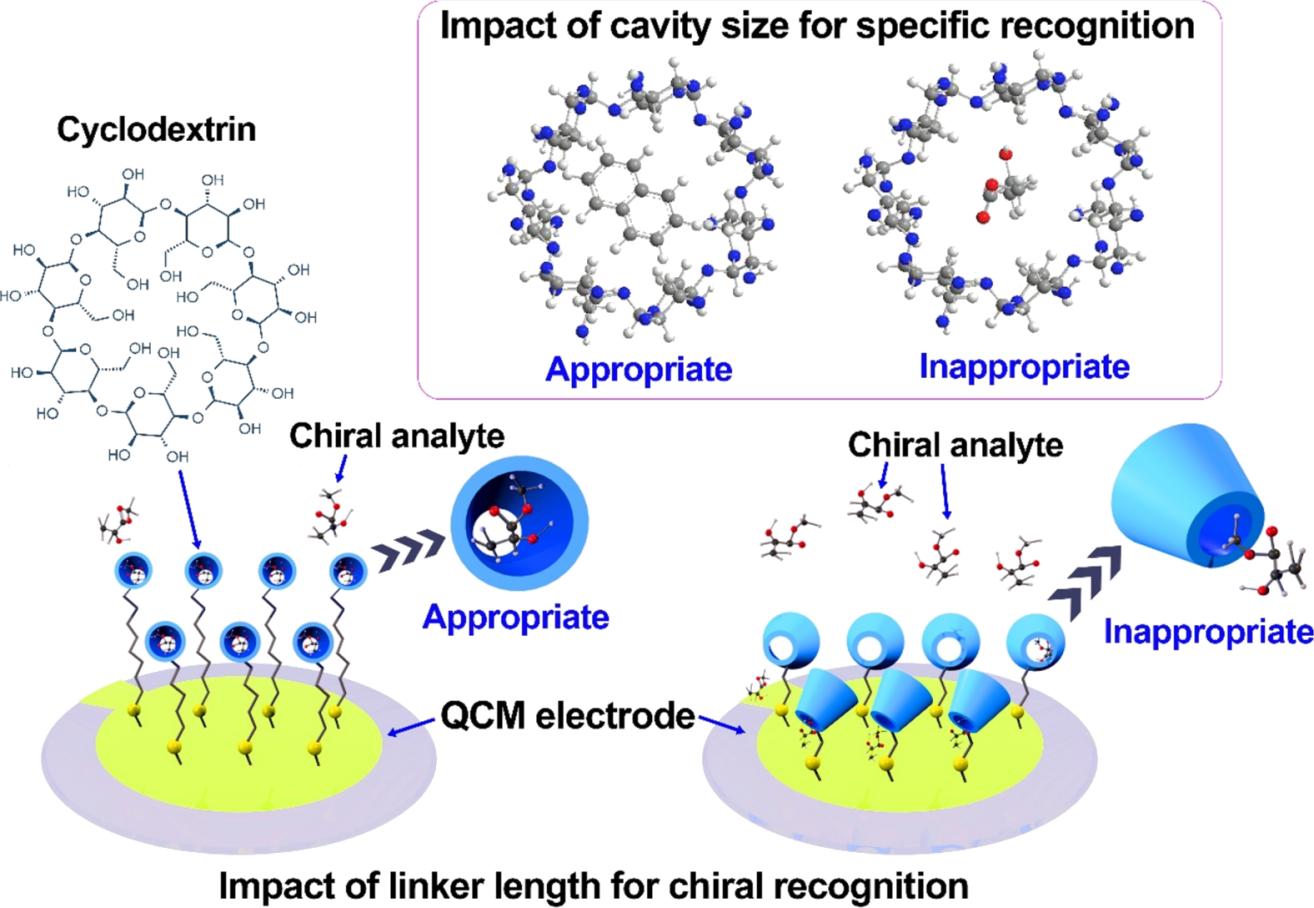
Chiral recognition of cyclodextrins by cavity size and linker length in the QCM system. The models illustrating the impact of cavity size on molecular recognition were adapted with permission from [69], Copyright 2008 American Chemical Society. This content is not subject to CC BY 4.0.

Based on the synthesis of mercaptyl-functionalized β-CDs with different linker lengths and functional groups on CDs, Xu et al. studied real-time chiral recognition of CD films to isomers in the gas phase [69]. Based on atomic force microscopy (AFM) observations, functional β-CDs with a short sulfide group were inclined to form monolayers. In contrast, those with long sulfide groups produced a quasi-two-layer packing with denser packing and higher surface concentration of CDs. The β-CD film with a long sulfide group thus exhibited better chiral discrimination capability (Figure 5). The cavity size of the functionalized β-CD may also affect the host–guest interactions and chiral discrimination (Figure 5). The cavity of native β-CD ranges from 0.60 to 0.65 nm and matches well with biphenyl or naphthalene groups, but not with methyl lactate, ethyl lactate, or 2-octanol. Conversely, β-CD with phenylcarbamate (Ph-β-CD) and 4-methoxyphenylcarbamate groups (MP-β-CD) possess smaller cavities and more rigid structures, which may lead to the preferential binding of small guests and higher chiral discriminating factors. Besides cavity size or shape fitting, regulation of noncovalent interactions for the recognition processes is also essential. Enantiomers may interact with the host in many ways but show a significant difference in the binding with a certain group of CDs. For example, the hydroxy group of (+)-methyl ʟ-lactate interacts with the methoxy group of MP-β-CD through hydrogen bonding. However, (−)-methyl ʟ-lactate does not undergo similar interactions due to the orientation difference of the hydroxy group. Therefore, the methoxy group, a hydrogen acceptor located at the external binding sites, plays a key role in improving chiral recognition. These studies suggest that a good design of cooperative weak interactions between host and guest molecules is needed to achieve better discrimination sensibility.

**Calixarenes.** Chiral calixarenes are a class of important host compounds which have wide applications in chiral recognition, enantiomer separation, and asymmetrical catalysis [70–72]. Calix[4]arene compounds are the most investigated molecules due to their stable bowl-shaped conformation and easy derivatization. The intramolecular cavities of calixarenes are known to be able to selectively interact with structurally complementary molecular species for molecular recognition purposes.

**Figure 6:**
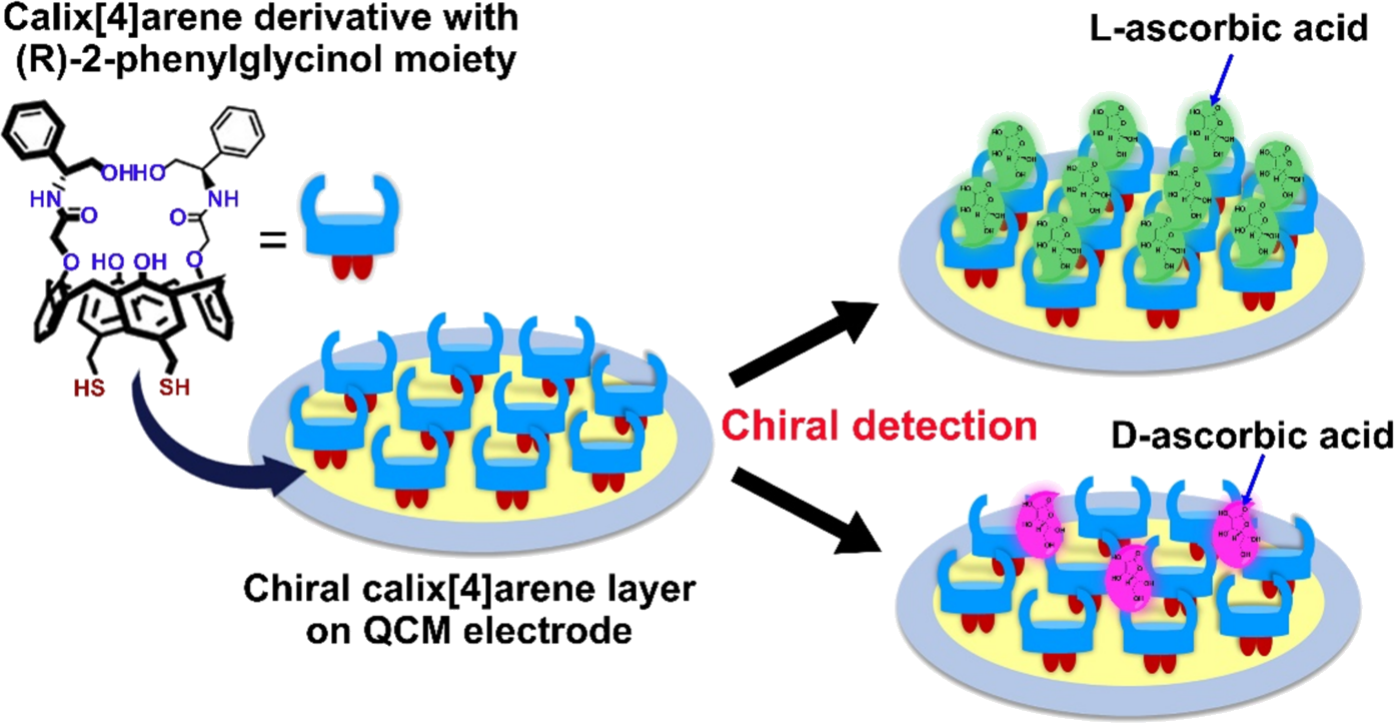
The chiral calix[4]arene layer as a selector for enantioselective adsorption of ascorbic acid in the QCM system. Redrawn from [76].

Chirality in calixarenes may derive from binding (at least) one chiral subunit to the rims or asymmetric placement of achiral subunits on the macrocycle [73–75]. Akpinar et al. reported a simple and quick chiral discrimination strategy for ascorbic acid (AA) enantiomers based on calixarenes films (Figure 6) [76]. A chiral calix[4]arene-bearing chiral phenyl glycinol moiety on the lower rim and a thiol moiety on the upper rim were deposited on the gold surface of the QCM electrode. The chiral calixarene film exhibited good and more than doubled selectivity towards ʟ-AA. Different factors may affect the sensing performance, including the “lock-and-key principle”, size-fit concept, three-dimensional structures of molecules, steric effects, and complex interactions between moieties of the sensor layer and analytes. To study the influence of chiral moieties of calixarenes, Akpinar et al. synthesized a series of chiral calix[4]arenes bearing chiral amine and amino alcohol moieties on their upper rim and a disulfide moiety on the lower rim [77]. The chiral calix[4]arene films can easily and stably coat the QCM electrode through sulfide interaction. It was found that the calix[4]arene derivative with (*R*)-2-phenylglycinol moieties has the best chiral detection efficiency for alanine. This system showed outstanding sensing properties, with real-time, sensitive and selective chiral detection, high durability, and easy recovery.

To understand the chiral recognition between calixarenes and analytes, Zoya I. Kazantseva et al. combined QCM and proton magnetic resonance spectroscopy (^1^H NMR) techniques to study the complexation process of various chiral calix[4]arenes derivatives with gas-phase *R*/*S*-1-phenethylamine [78]. They proved that due to the acid–base interaction, only derivatives with acid could bind well with chiral amines. The other derivatives with esters, amides, and alcohols showed low sensitivity to 1-phenylethylamine. The best enantio-binding properties were shown for monopropoxy-calix[4]arene acetic acid. It was also found that the position of the propoxy group in the dipropoxy-calix[4]arene acetic acid may greatly change the chiral selectivity to 1-phenethylamine. The *meta*-position promoted the combination of *S*-1-phenethylamine, while the *ortho*-position may combine with *R*-1-phenethylamine. The QCM results were consistent with the data from ^1^H NMR spectroscopy. Moreover, the QCM method may also allow for the detection of weak interactions of amides and esters with amines and a slight enantiodiscrimination.

**Porphyrin derivatives.** Porphyrins and metalloporphyrins have been proposed to be suitable hosts for chirality sensing due to their functionalization at peripheral positions of the framework and easily monitoring of structural changes by strong optical absorptions [79–81]. They may also be used as building blocks for organized materials expressing chirality at the supramolecular level [82–83].

The introduction of metal centers in porphyrins could further enhance the recognition interactions and form a more stable host–guest complex for chiral sensing [84]. For example, Simonneaux et al. reported a Ru-modified porphyrin as a chiral recognition host to achieve specific recognition of racemic isocyanides and alcohols [85]. Imai et al. and Hayashi et al. obtained chiral recognition of amino acids and peptides using Zn-modified porphyrins [86–87]. In general, the chirality of the porphyrin-based supermolecular systems is generated either via the intrinsic chiral modification of achiral porphyrinoids or via the external chiral field.

**Figure 7:**
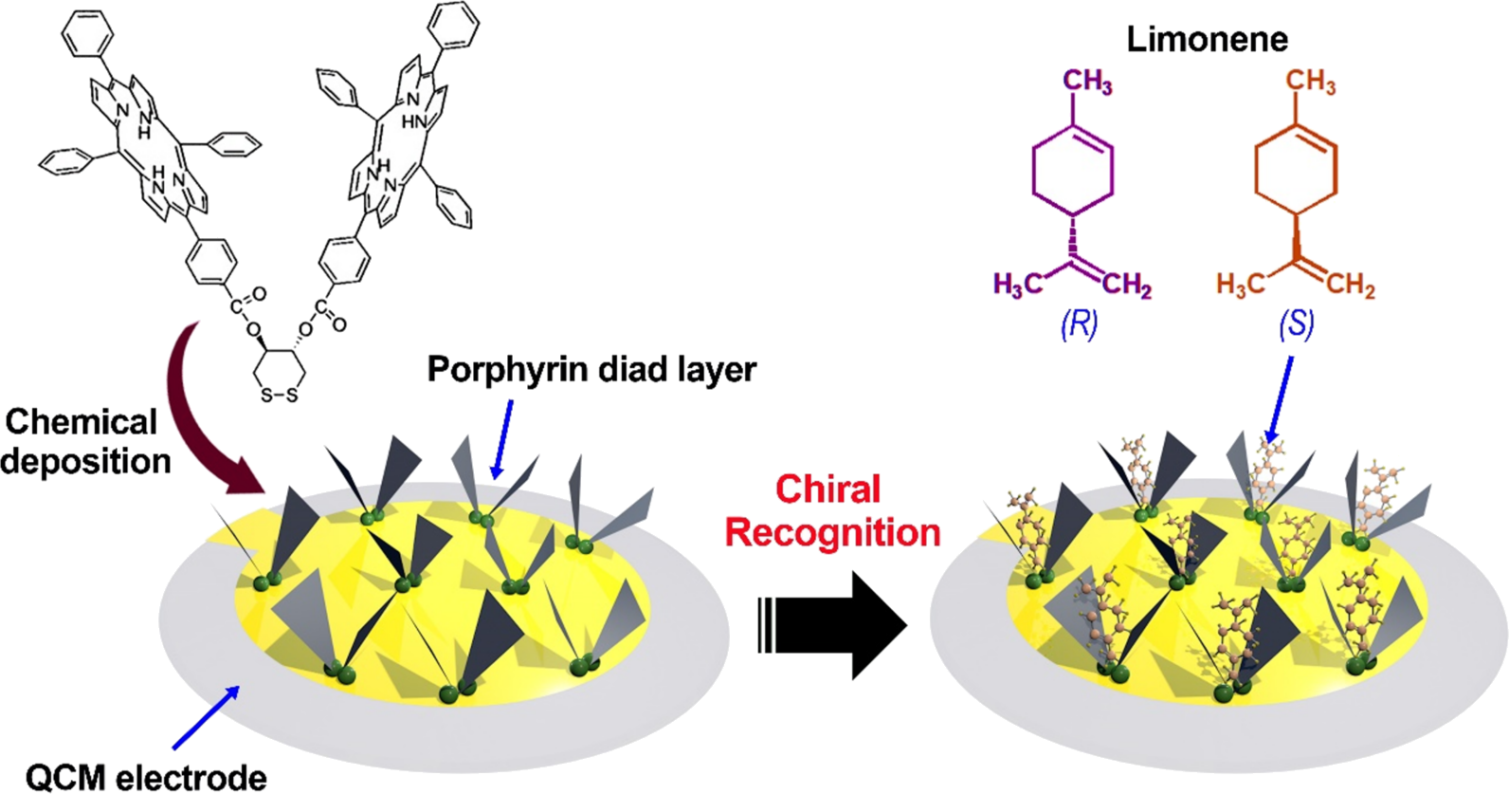
The porphyrin diad layer as a chiral selector for detection of chiral limonene in the QCM system [88].

Supramolecular porphyrin films were also successfully used in QCM-based chirality-sensing systems. Paolesse et al. deposited a porphyrin diad layer as the chiral receptor for gas-phase detection of chiral compounds (Figure 7) [88]. The gold surface of the electrode was first modified with *trans*-1,2-dithiane-4,5-diol, then a monolayer of [Co_2_(porphyrin diad)] complex with the porphyrin rings oriented in an almost perpendicular fashion to the surface was adsorbed. Based on this system, significant chiral discrimination was observed for limonene with a binding stoichiometry of about 1:3 for *S*-limonene. Combined with UV–vis spectrometry analysis results, it was shown that the chiral recognition was based on the formation of a sandwiched host–guest complex by a π–π interaction between limonene double bonds and the aromatic system of two porphyrin rings. The overall association constant values *K* of *S*-(−) over the *R*-(+) limonene have a big difference, which was 6200 and 1600 m^−1^, respectively. These results thus provided a novel detection system for chiral volatile organic compounds (VOCs) with a remarkable degree of selectivity, which may promote the development of electronic nose systems for chiral analytes.

**Metal–organic frameworks.** Metal–organic frameworks (MOFs) are unique porous crystalline materials fabricated by the self-assembly of metal ions or clusters and organic ligands via coordination bonds [89–92]. The variety of combinations between metal ions and organic linkers or structural motifs allows for tunable pore size/shape and adjustable surface functionality [93–94]. These structural characteristics make MOFs one of the most ideal sensing materials [95–97].

Due to the confinement effect from the porous space, chiral MOFs with suitable recognition sites may improve the stereoselectivity of chiral sensing [98]. Zhu et al. designed a homochiral MOF sensor based on [Zn(L)(2,2′-bipy)]·H_2_O, which could achieve the quantitative enantioselective sensing of chiral amino acids [99]. The multiple noncovalent interactions, including metal coordination, hydrogen bonding, and π–π stacking between individual chiral amino acids and the MOF sensor enabled accurate determination of the absolute configuration and enantiomeric ratio of the target amino acids in the presence of other amino acid interferences. The resultant sensing system has also the advantages of being simple, reusable, and can be easily manipulated for enantioselective sensing assays.

**Figure 8:**
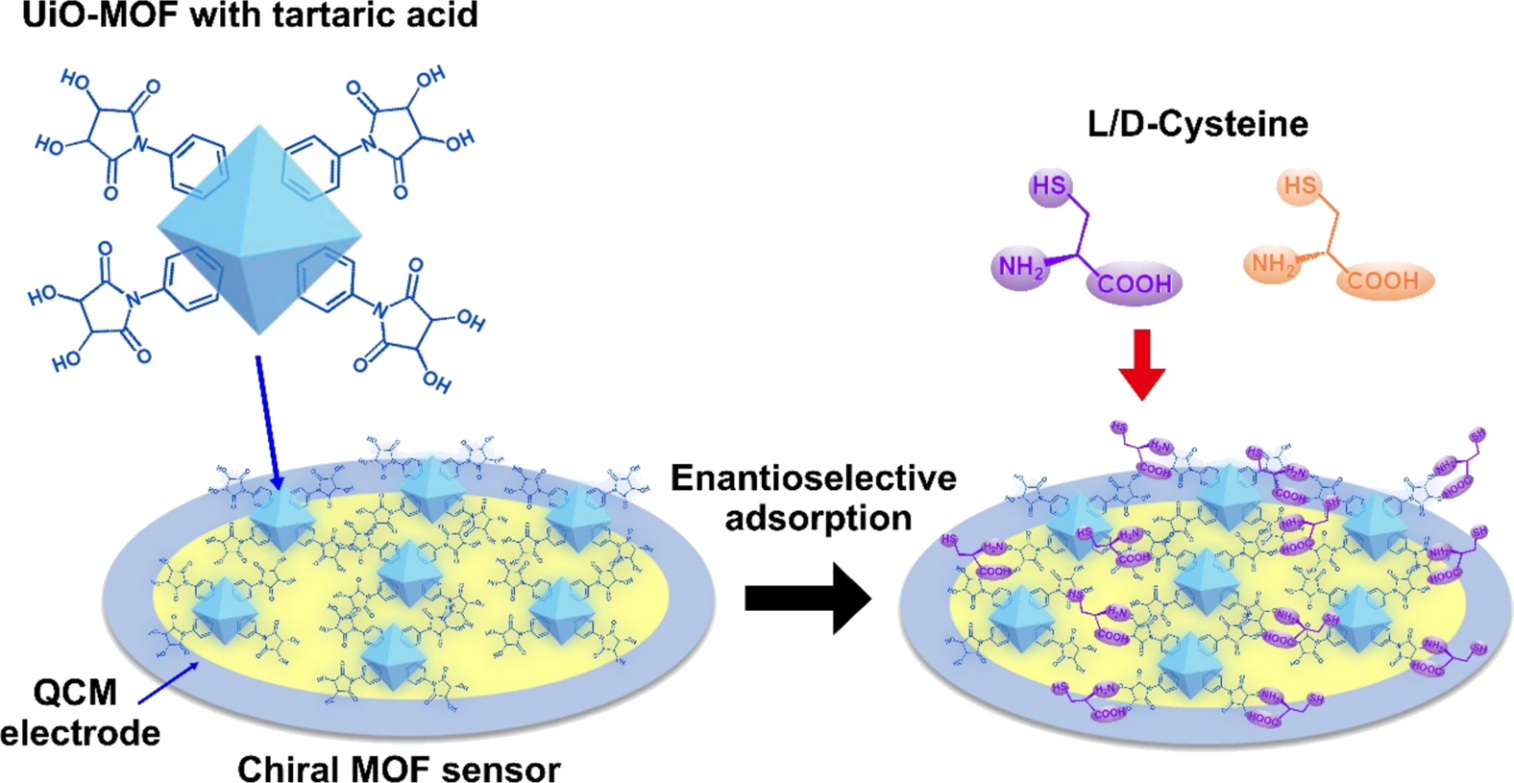
The UiO-MOF-derived QCM sensor for efficient discrimination of cysteine enantiomers in the QCM system [100].

Rational selection of chiral ligands is the key factor for successful chirality detection by MOFs. Yang et al. reported the fabrication of chiral UiO-MOF-derived QCM sensors for efficient discrimination of cysteine (Cys) enantiomers (Figure 8), of which the ʟ-type is vital in biological processes but the ᴅ-type has a hazardous effect [100]. The chiral UiO-MOF sensors (ʟ- and ᴅ-UiO-tart) were facilely prepared by post-modification of UiO-66-NH_2_ with chiral tartaric acids and coated on the surface of the QCM electrode as a chiral selector for enantioselective adsorption of a specific Cys enantiomer. This sensing system showed highly enantioselective activity and an enantioselective factor of up to 5.97 ± 0.54, which was the best performance of MOFs for Cys enantiomer discrimination.

Xylene is an important chemical feedstock and has three isomeric forms: *o*-xylene, *m*-xylene, and *p*-xylene. Although they have similar structures and physical properties, the three xylene isomers have different metabolic pathways in humans and other mammals [101]. Heinke et al. identified xylene isomer mixtures by using QCM-based sensors coated with selected MOF films with different isomer affinities [102]. The sensor was composed with six MOF films of Cu_3_(BTC)_2_ (BTC = benzene-1,3,5-tricarboxylate), Cu(BDC) (BDC = benzene-1,4-dicarboxylate), Cu(BPDC) (BPDC = biphenyl-4,4′-dicarboxylate), UiO-66, UiO-67, and UiO-68-NH_2_. The sensing system showed a very low detection limit of 1 ppm for each pure isomer and can discriminate between 16 different xylene mixtures with 96.5% accuracy at a concentration of 100 ppm. Molecular simulation results indicated that the isomer discrimination is mainly due to the access of the isomers to different adsorption sites in the MOFs, which are sterically controlled by the rigid crystalline framework.

**Figure 9:**
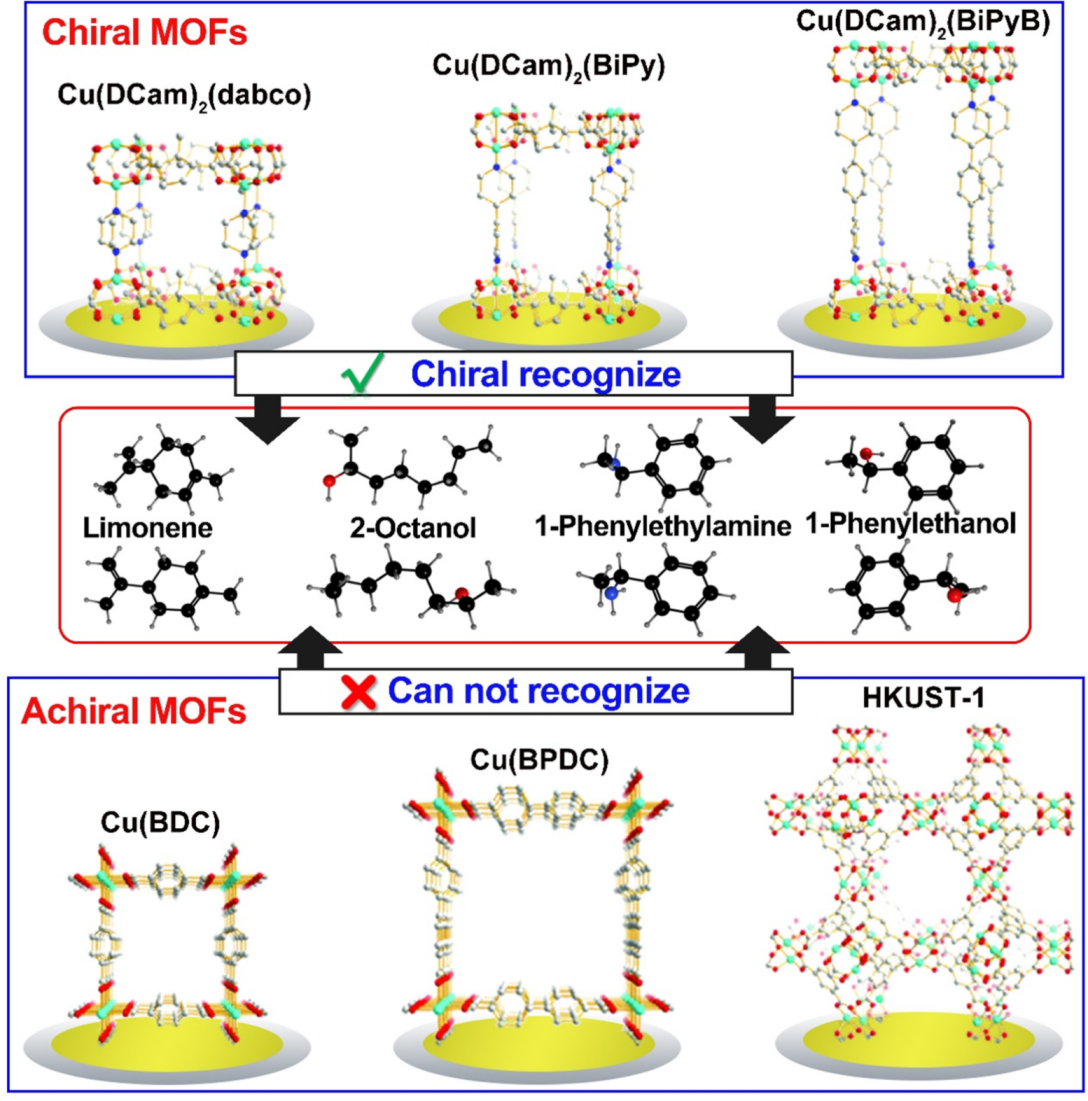
The comparison of homochiral and achiral MOF structures for chiral recognition in the QCM system. Redrawn from [103].

Based on the same detection mode, the QCM-based sensor array coated with six different MOF structures was further constructed, comprising three homochiral and three achiral structures (Figure 9) [103]. The homochiral MOFs were Cu_2_(DCam)_2_(dabco), Cu_2_(DCam)_2_(BiPy), and Cu_2_(DCam)2(BiPyB), in which DCam = d-camphorate, dabco = 1,4-diazabicyclo[2.2.2]octane, BiPy = 4,4′-bipyridyl, and BiPyB = 1,4-bis(4-pyridyl)benzene. The achiral MOF structures were Cu_3_(BTC)_2_, Cu(BDC), and Cu(BPDC). The QCM sensor array successfully worked as an electronic nose system for detecting chiral odor molecules of limonene, 2-octanol, 1-phenylethanol, 1-phenylethylamine, and methyl lactate. The achiral MOF structures showed very similar responses for isomers and could not distinguish different molecules, while the homochiral MOF structures could enantioselectively distinguish chiral molecules. The combined capability of the sensor array allowed for the enantioselective detection and discrimination of chiral odor molecules with 96% accuracy.

Besides regulating the porous structure and functionalities of MOFs, it may also improve the chiral sensing performance by controlling the orientation growth of MOFs to the assembled composite films on the QCM surfaces. Wöll and Fischer et al. reported the construction of chiral MOF-based films by layer-by-layer (LBL) liquid-phase epitaxial (LPE) growth of [{Zn_2_((+)cam)_2_(dabco)}*_n_*] ((+)cam = (1*R*,3*S*)-(+)-camphoric acid, dabco = 1,4-diazabicyclo[2.2.2]octane) [104]. Through using SAMs with carboxylate and pyridyl groups, composite films (SURMOFs) with the enantiopure [{Zn_2_((+)cam)_2_(dabco)}*_n_*] oriented in the (110) or (001) direction, were obtained and showed significant enantioselectivity.

Duan et al. fabricated 3D chiral porous Zn-organic frameworks (Zn_2_(bdc)(ʟ-lac)(dmf)-DMF) (ʟ-lac = ʟ-lactic acid, bdc = 4-benzenedicarboxylic acid) on the Au electrode surface of QCM for chiral discrimination of four pairs of enantiomers [105]. The Zn_2_(bdc)(ʟ-lac) (dmf)-DMF has an open architecture, whose pores are interconnected in three directions. The ʟ-lactate moieties in Zn_2_(bdc)(ʟ-lac)(dmf)-DMF offer the chiral centers within the voids and provide a homochiral environment. The sensor showed excellent sensitivity and enantioselectivity to ʟ-phenylethylamine, whose recognition ability was temperature- and concentration-dependent. The variation in the chiral selectivity factors was suggested due to the matching of molecular size to the channel of the MOFs.

### Chiral sensing layers from inorganic nanostructures

In contrast to the abundance of chiral organic molecules, chirality in inorganic materials seems rare. Unlike the well-established theory of chirality for organic molecules, the notion of chirality for metals, semiconductors, and other inorganic nanostructures is still evolving [106–107]. The reason for this is that in inorganic nanostructures there are multiple symmetric and asymmetric relations in the geometry of their atoms. The chirality of inorganic materials may originate from space chiral ligands, the shape of the inorganic core, chiral surfaces, and the tetrahedral geometry of atomic packing in many nanoscale crystals [108–110]. The chiral inorganic nanostructures thus may create an asymmetric environment for enantiomers. As inorganic materials possess higher stability and might be suitable for different types of enantiomers, they are considered to be the most promising platform for enantiospecific sensing and may also be explored for chiral separation and catalysis [111].

#### Induced chiral metal or inorganic oxides

Based on the template strategy, metals or inorganic oxides with chiral channels could be fabricated by using chiral templates [112–113]. Such materials possess controllable pore dimensions, compositions, and high surface areas, which may be intriguing substrates for chiral catalysis, sensing, and separation of various kinds of chiral analytes [114–115]. For example, Qiu et al. used SiO_2_ with chiral channels obtained after chiral anion induction and combined with CD spectroscopy to achieve chiral recognition with a relatively larger size of poly(propiolic acid) sodium salt [116]; He et al. used chiral mesoporous SiO_2_ and combined with gas chromatography (GC) achieved chiral separation of amino acid derivatives and other substances [117].

Various metal or inorganic materials have been explored as chiral selectors in QCM systems based on different strategies and techniques. Sarkar et al. used tartaric acid as a chiral inducer to control the crystalline orientation of CuO films based on the electrodeposition method [118]. The deposited CuO film onto highly symmetrical Au(111) surfaces was shown to have mirror-symmetric chirality. The enantiospecificity of the films was studied using QCM and evaluated according to the changes by the selective oxidation of chiral tartaric acid. The films etched in ʟ-(+)-tartaric acid were shown to prefer the ʟ-(+)-tartrate oxidation, whereas the etched films in ᴅ-(−)-tartaric acid tend to oxidize ᴅ-(−)-tartrate. This indicated that the produced chiral surfaces of the CuO films from the etching process may regulate the chiral selective reactions on the surface.

Jie et al. synthesized chiral *R*/*L*-TiO_2_ nanofibers by using *N*-stearoyl- ʟ/ᴅ-glutamic acid (C18-ʟ/ᴅ-Glu) lipid as the chiral template and titanium diisopropoxide bis(acetylacetonate) as the titanium source based on a sol–gel process [119]. The average length and width of the TiO_2_ nanofiber were ca. 200 and 30 nm, respectively. The resultant *L*- or *R*-TiO_2_ nanofibers were immobilized as recognition layers on the QCM electrode for selective adsorption of chiral peptides. The insulin monomers exhibited a higher tendency to bind to the *R*-surface than to the *L*-surface. The recognition difference was suggested mainly due to the steric effect between the interaction of the amino acid residues of insulin and the O^−^ and OH^2+^ groups on TiO_2_. The helical arrangement of the nanoscale lattice planes of TiO_2_ in the *R*-surface may provide a right-handed helical structure, leading to strong interaction with the insulin monomer. The *R*-surface thus can retain the initial orientation and the natural activity of the insulin molecules. Conversely, the weak interaction of the insulin monomer on the *L*-surface makes it hard to maintain its initial helical arrangement and it only unfolds to form the oligomer.

**Figure 10:**
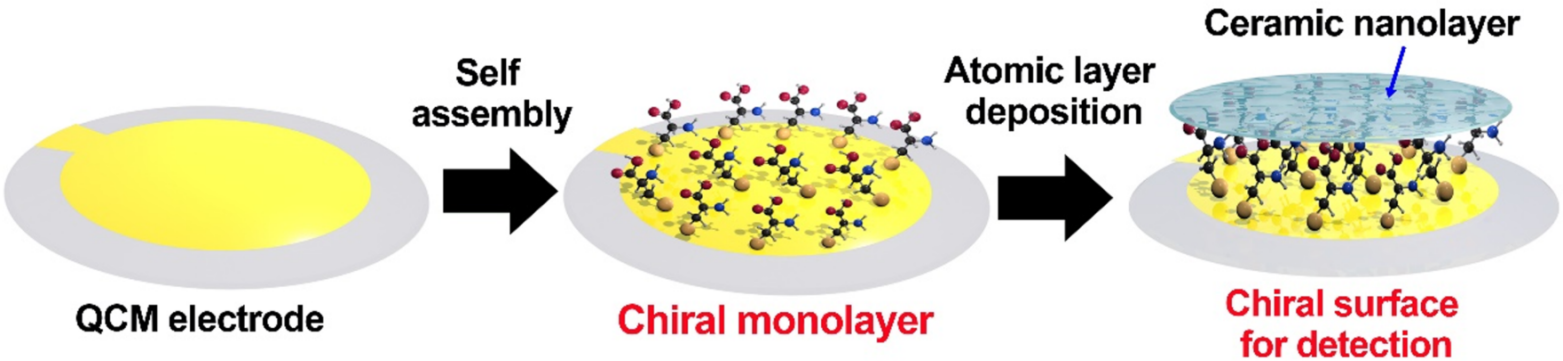
The formation of a chiral ceramic layer for chiral recognition in the QCM system [121].

Moshe et al. fabricated chiral TiO_2_ films of [Ti{N(CH_3_)_2_}_4_] on SAMs of chiral molecules by using the atomic layer deposition (ALD) technique [120]. The specific selection effect was verified by QCM measurements using valine (Val) as the target analyte. The TiO_2_-SAMs films were shown to preferentially adsorb ᴅ-Val, suggesting a reliable chiral selector structure. The research group also prepared chiral Al_2_O_3_ films by a similar process using SAMs of cysteine (Cys) and KAl_2_(AlSi_3_O_10_)(OH)_2_ precursors (Figure 10) [121]. The resultant Al_2_O_3_ films were efficiently deposited on the SAMs and formed a very smooth and conformal film with a thickness of (8.9 ± 0.1) nm. The QCM measurements exhibited a 690 ng/cm^2^ difference between the adsorption amount of ᴅ- and ʟ-tartaric acid, and it proved the enantioselective adsorption on the chirality imprinted Al_2_O_3_ films.

Yemini et al. reported the fabrication of Zn/Cys chiral nanostructures by molecular layer deposition (MLD) using diethylzinc (DEZn) as the inorganic precursor and Cys enantiomer as the chiral organic precursor [122]. The Zn/Cys nanostructures showed a size of 15 nm and could tightly aggregate into a homogeneous and continuous film on the QCM surface. The QCM adsorption results indicated that ʟ-alanine was 307.97% more enantioselective than ᴅ-alanine. Similar enantioselectivity could also be achieved by Al/ʟ-Cys MLD films [123]. The methodology is also versatile and promising for the deposition of chiral thin films over any substrates or membranes of interest, which may promote the exploration of metal-based chiral sensing applications.

Based on surface modification or induced crystallization, metal nanostructures may bear chiral surfaces for chiral sensing of enantiomers. For example, Zhang et al. achieved chiral recognition of cysteine enantiomers using nucleotide-modified Ag nanoparticles [124]. Zhang et al. used ʟ-cysteine modified Au nanoparticles for chiral recognition of carnitine [125]. Jafari et al. used chitosan modified Ag nanoparticles for chiral sensing of tryptophan enantiomers [126]. Niu et al. reported the chiral recognition of tryptophan (Trp) on chiral Au facets generated by polypyrrole (PPy) and Trp [127].

#### Intrinsic chiral metal crystals

Until relatively recently, metals were ignored as potential substrates for asymmetric surface chemistry since metals always show highly symmetric and achiral bulk structures with unexposed chiral surfaces [128–129]. Sykes and co-workers demonstrated that the metals with high Miller index surfaces M(*hkl*) (*h* × *k* × *l* ≠ 0 and *h* ≠ *k* ≠ *l* ≠ *h*) can show chirality and exist in two enantiomeric forms: *R-* and *S*- [130]. Some metallic alloys and inorganic compounds such as PdGa, Te, and HgS may also possess bulk chiral crystal structures and expose chiral surfaces even with high symmetry and low Miller index surface orientations [131–133]. Therefore, metals with chiral surfaces may have enantiospecific interactions with chiral molecules [134–138].

Scanning tunneling microscopy (STM) studies and simulations indicated that metal surfaces may be an ideal platform to study the dynamic chiral recognition process. The adsorption orientations of the two enantiomers may have clear differences on the chiral metal surface, which makes the binding energy of enantiomers different on the crystalline surface. Dong and Wang et al. proved by STM the molecular-level identification of a chiral recognition process of phthalocyanine (Pc) on a Cu(100) surface [139]. They revealed the critical role of the particular adsorption geometry on the metal surface in the chiral-specific configuration of Pc. Kong and co-workers successfully controlled the long-range chirality recognition of 3-bromonaphthalen-2-ol (BNOL) on a Au(111) surface and proved the recognition force from the herringbone reconstruction-induced accumulation of dipoles at the stripe edge of BNOL [140]. Liu and Li et al. studied the enantiospecific adsorption of α-amino acids on metal crystals of Ag, Cu, Pt and alloys by density functional theory (DFT) simulations. They revealed that Pt(531) with a step–kink metal surface has better enantiospecificity for eight α-amino acids (alanine, α-aminobutyric acid, valine, leucine, phenylalanine, serine, cysteine, and 3-aminoalanine) [141–142]. The step–kink structure can provide more adsorption sites for chiral molecules, thus promoting the separation of the two isomers.

**Figure 11:**
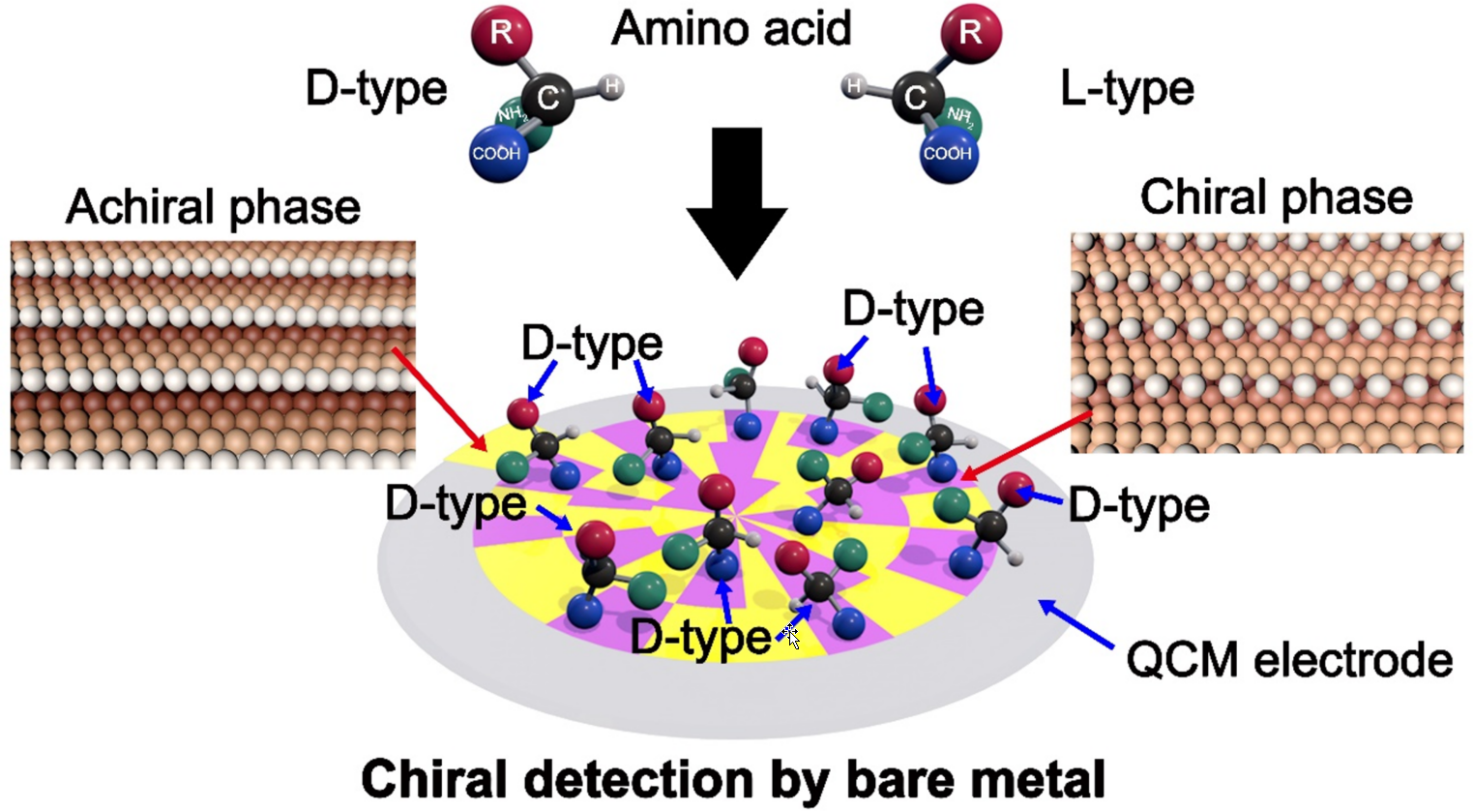
The chiral recognition by a bare metal layer in the QCM system. Redrawn from [144].

Although based on simulation studies, the chiral metal surface may induce different conformational strains with enantiomers and exhibit distinct adsorption energies [143]. Bare metal layers were very rarely used for chiral recognition directly in the QCM system. Ji and Liu et al. studied for the first time the chiral adsorption behavior of bare gold electrodes for amino acids by QCM (Figure 11) [144]. The Au surfaces contain 27–37% of chiral surfaces and the most exposed surface of Au(324). The QCM adsorption in aqueous solutions of amino acids showed that ᴅ-type amino acids prefer to adsorb on the surface. The DFT calculations further proved that the enantioselective adsorption in this system is probably caused by the existence of chiral planes in the metal. Different from all prior QCM-based chiral sensing systems, no organic coating/recognition layer was used in this case. With this approach, dissipation of the oscillation or detachment of the organic selector layers is avoided, and the signal transfer during the molecular recognition processes is strengthened. This work presented the great potential of bare metal surfaces as an effective platform for chirality detection.

**Figure 12:**
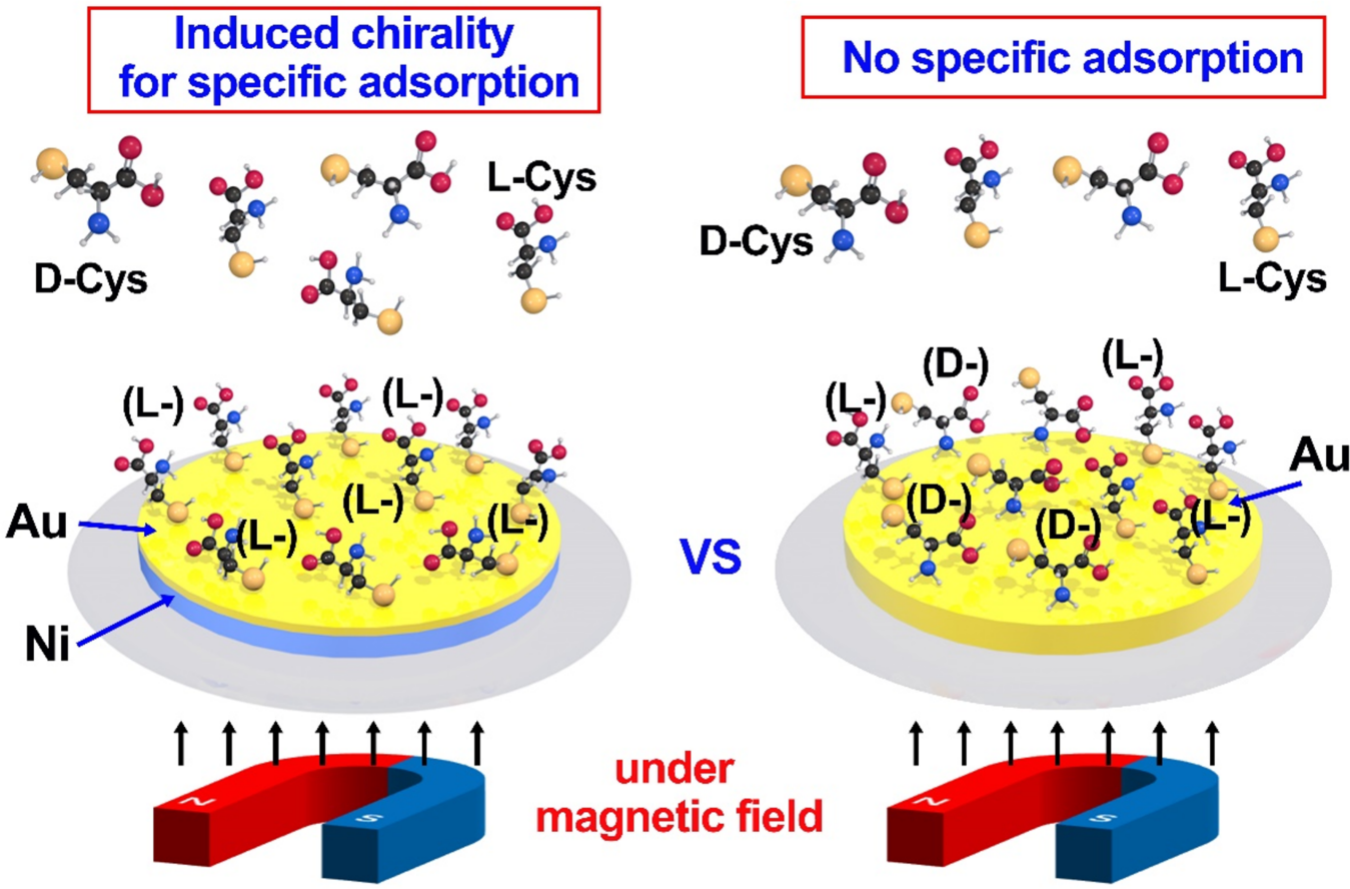
The chiral recognition on the metal layer induced by magnetic field in the QCM system [145].

Metals are not only more stable than organic materials in various usage conditions, but also have excellent optical, electrical, and magnetic properties. They may facilitate the design of novel chiral sensing systems from the viewpoints of structural features and functionalities. The induced changes of the electron spin orientation in metals may cause charge redistribution in chiral molecules and manifest an enantiospecific preference. Paltiel and Naaman et al. first elucidated that the induced spin polarization may affect enantiorecognition under an external field. They experimentally showed that the interaction of chiral molecules with a perpendicularly magnetized substrate was enantiospecific (Figure 12) [145]. The spin-specific interactions between magnetic metals and chiral molecules may allow for the separation of enantiomers such as oligopeptides.

Y. Lu et al. investigated the enantiospecific interaction between a magnetized surface and a chiral amino acid by using electrochemical quartz crystal microbalance (EQCM) and Cys as a chiral model [146]. The enantiospecific adsorption on a ferromagnetic Ni surface was proven to arise from the adsorption kinetics rather than from thermodynamic stabilization. The adsorption rate of ᴅ-Cys and ʟ-Cys onto a ferromagnetic substrate showed a significantly different behavior according to the magnetic field direction and molecular handedness.

#### Chiral modified carbons

Carbon nanomaterials possess attractive features since they are low cost, capable to be produced in large-scale, and have good stability and bio-compatibility, which makes them an excellent candidate for sensing applications [147–149]. Some carbon nanostructures such as carbon nanotubes and fullerenes were demonstrated to have chirality. However, the preparation of chirality-pure substrates still requires the combination of specific carbon nanostructures and homochiral functionalizations [150–151].

Protein misfolding, which may form amyloid aggregates, is the main cause of neurodegenerative diseases. Qing et al. used a chiral cysteine- (*L*/*R*-Cys) modified graphene oxide (GO) to study the chirality of the aggregation process of the chiral amyloid β-protein(1-40) (Aβ(1-40)) [152]. The adsorption behaviors of Aβ(1–40) monomers and oligomers by QCM showed that GO significantly promoted adsorption, indicating the crucial role of a GO surface in the chiral effect. Combined with other analysis techniques, it proved the strong influence of surface chirality on the conformational transition from α-helix to β-sheet, the adsorption of monomers and oligomers, and the subsequent fibrillation process. The results give interesting insights into the crucial roles of biological membranes on protein amyloidosis, and how intrinsic chirality contributes to this process. It also brings the prospect of chiral-modified carbon nanostructures for biological and medical applications.

## Conclusion

Quartz crystal microbalance provides one of the most versatile sensing technologies due to its low cost, rapid response, high sensitivity, easy operation, and real-time detection in contrast to conventional assay systems. It is one of the most important instruments for constructing novel chiral sensors and studying their recognition mechanisms. The sensitivity and specificity of QCM-based chiral sensors are dependent on the recognition layers on the electrode surface. Various strategies have been developed for the specific adsorption of enantiomers based on different nanomaterials and nanostructures. Especially, self-assembled supermolecular nanostructures provide more feasibility to regulate the detection interactions, which makes the sensing system effective for a wide range of chiral analytes.

Although most QCM-based chiral sensors are based on organic layers, inorganic materials or nanostructures are also very promising selectors from the viewpoints of better stability, diverse physical properties, and relatively easy and scalable synthesis. However, the reports about the usage of solely inorganic or metal layers as selectors are very rarely in QCM systems because of the lack of intrinsic chirality and unclear specific adsorption mechanisms. Therefore, constructing effective inorganic selectors in the QCM system requires more breakthroughs and developments in the fabrication of chiral inorganic or metal nanostructures. With other combined advanced equipment, the chiral detection mechanisms also need to be further elucidated by including analyses at atomic or molecular levels.

At present, other advanced analytical techniques such as GC, CD, and electrochemical measurements could also offer advantages for the separation or detection of chiral molecules. As QCM may give a high sensitive mass response on adsorption, it may be suitable for both separation and detection usages at the same time when employing proper sensing layers. Recently, there have been also advances in QCM instruments, such as super-high resonance frequency with >100 MHz QCM and wireless electrode-free QCMs. This enables QCM systems to detect surface changes in the microscale with higher accuracy and to be more conveniently used for various biological sensing applications [153–157].

The combined analysis systems of QCM with surface plasmon resonance (SPR), FTIR, and electrochemical station may also further strengthen the recognition efficiency and understanding of detection behaviors. Although for most cases QCM sensors are measured under atmospheric pressure and at room temperature, they may also work in a wide temperature range of −40–150 °C to enable temperature-dependent behavior studies. Therefore, by exploring more controlled techniques on chiral nanostructures, QCM holds great promise and potential for studying chiral sensing behaviors and constructing the next generation of novel chiral sensors.
